# NK Cell Induced T Cell Anergy Depends on GRAIL Expression

**DOI:** 10.3390/cells8080790

**Published:** 2019-07-29

**Authors:** Grazyna Galazka, Malgorzata Domowicz, Alicja Ewiak-Paszynska, Anna Jurewicz

**Affiliations:** Department of Neurology, Medical University of Lodz, 22 Kopcinskiego str, 90–153 Lodz, Poland

**Keywords:** multiple sclerosis, experimental autoimmune encephalomyelitis, NK cell, anergy, GRAIL

## Abstract

NK cells (natural killer cells) being a part of the innate immune system have been shown to be involved in immunoregulation of autoimmune diseases. Previously we have shown that HINT1/Hsp70 treatment induced regulatory NK cells ameliorating experimental autoimmune encephalomyelitis (EAE) course and CD4+ T cells proliferation. NK cells were isolated from mice treated with HINT1/Hsp70 and co-cultured with proteolipid protein (PLP)-stimulated CD4+ T cells isolated from EAE mice. Cell proliferation was assessed by thymidine uptake, cytotoxicity by lactate dehydrogenase (LDH) release assay and fluorescence activated cell sorting (FACS) analysis, protein expression by Western blot, mRNA by quantitative RT-PCR. Gene related to anergy in lymphocytes (GRAIL) expression was downregulated by specific siRNA and GRAIL overexpression was induced by pcDNA-GRAIL transfection. HINT1/Hsp70 pretreatment of EAE SJL/J mice ameliorated EAE course, suppressed PLP-induced T cell proliferation by enhancing T cell expression of GRAIL as GRAIL downregulation restored T cell proliferation. HINT1/Hsp70 treatment induced immunoregulatory NK cells which inhibited PLP-stimulated T cell proliferation not depending on T cell necrosis and apoptosis. This immunoregulatory NK cell function depended on NK cell expression of GRAIL as GRAIL downregulation diminished inhibition of NK cell suppression of T cell proliferation. Similarly GRAIL overexpression in NK cells induced their regulatory function. HINT1/Hsp70 treatment generated regulatory NK cells characterized by expression of GRAIL.

## 1. Introduction

The cells of an adaptive immune system, in contrary to cells of an innate immune system, have been regarded as cells creating immunological memory after an initial response to a specific pathogen leading to an enhanced response to subsequent encounters with that pathogen. However, increasing evidence has been gathered showing that cells belonging to innate immune system have the ability to regulate adaptive immune response and express features of an immunological memory [1]. NK cells being a part of the innate immune system have been implicated in autoimmune diseases as diabetes, rheumatoid arthritis, and multiple sclerosis [1]. Multiple sclerosis (MS) and its animal model—experimental autoimmune encephalomyelitis (EAE)—have been characterized by inflammation within central nervous system represented by immune cell infiltration, demyelination, and axonal damage, resulting in clinical manifestation of the disease. Immune cells infiltrations observed in multiple sclerosis lesions contain monocytes/macrophages, microglia, T lymphocytes (CD4+, CD8+, and γδ) and NK cells [2]. T lymphocytes are regarded as main immune cells responsible for pathogenic changes in MS [3].

T cell anergy is a tolerance mechanism in which the lymphocyte is intrinsically functionally inactivated following an antigen encounter, but remains alive for an extended period of time in a hyporesponsive state [4]. It might be induced by lack of costimulation, altered peptide ligands with lower avidity of TCR ligation resulting in decrease T cell proliferation and cytokine production [4,5]. Several negative regulatory factors including Cbl-b, c-Cbl and Itch, Egr2, DGK-α, Lag3, and gene related to anergy in lymphocytes (GRAIL) were indicated to be involved in induction of T cell anergy [6,7,8,9,10]. GRAIL is a well-studied regulator involved in the induction and maintenance of T cell anergy [11]. However, the function of GRAIL in other immune responses is not well known. Several other E3 ligases that regulate T cell anergy, such as Cbl-b, c-Cbl, and Itch, have been demonstrated to have important roles in the regulation of innate immunity, including function of macrophages [12,13]. In addition, GRAIL has been reported to mediate p53-dependent cell cycle arrest and apoptosis by targeting this protein for degradation [14]. However, there is no data on GRAIL function in NK cells.

The role of NK cells in multiple sclerosis was highly investigated when effective treatment with daclizumab (an antibody against the IL-2 receptor α subunit, CD25) was correlated with increase of CD56^bright^ NK cell population in peripheral blood [15,16]. Similarly other MS therapies as interferon β and natalizumab (an antibody against adhesion molecule VLA-4) showed enhancement of CD56^bright^ NK cell population in peripheral blood [17,18]. These findings are consistent with the suppressive effect of the CD56^bright^ NK cells observed in MS and their lytic activity against activated (but not resting) CD4+ T cells [19,20]. This finding suggests a mechanism for NK cell suppressive activities in MS.

The role of NK cells in experimental autoimmune encephalomyelitis the animal model of MS is controversial. Depletion of NK cells before EAE induction augments clinical manifestation of disease [21,22,23,24]. Most of the data suggest that NK cells are important for EAE amelioration due to their cytotoxicity capacity directed to inflammatory cells (T lymphocytes, monocytes) [25]. In our previous experiments, we have shown that tolerogenic effect of NK cells stimulated with HINT1/Hsp70 depended on NKG2D stimulation and did not involve increased NK cell cytotoxicity to CD4+ T lymphocytes [26]. In our present experiments, we have shown that NK cells stimulated with HINT1/Hsp70 was characterized by increase GRAIL expression and these NK cells induced anergy in CD4+ T lymphocytes.

## 2. Material and Methods

### 2.1. Animals

Female SJL/J and C57BL6/J mice, 6 to 8 weeks of age, were obtained from The Jackson Laboratory (Bar Harbor, ME, USA), and all animal procedures were approved by the Animal Care and Use Committee of the Medical University of Lodz. The NK cells isolated from C57BL6/J mice were used in experiments with GRAIL transfection. The C57BL6/J mice are resistant to HINT1/Hsp70 induced inhibition of EAE as they do not express H60 which is indispensable for HINT1/Hsp70 induced tolerance. Therefore tolerance induction in these cells with GRAIL transfection confirms crucial function of this molecule in tolerogenic immune response.

### 2.2. Reagents

The following Abs were used: anti-Hsp70 mouse monoclonal Ab, anti-mouse IgG-HRP goat Ab conjugated with HRP, anti-GRAIL goat polyclonal Ab and anti-goat IgG-HRP Ab conjugated with HRP (Santa Cruz Biotechnology, Santa Cruz, CA, USA), rabbit anti-HINT1 (PRS4815, Sigma, St. Louis, MO, USA), and HRP–conjugated goat anti-rabbit Ab, (Santa Cruz Biotechnology). Rabbit anti-asialo GM1 antibody was used for in vivo experiments to delete NK cells (Wako, Osaka, Japan). Mouse Abs consisted of: R-PE or FITC-conjugated rat anti-mouse CD49 b/pan-NK mAb (DX5 Ab); R-PE, FITC or Per CP-conjugated anti-CD3ε mAb; Per CP-conjugated anti-CD4 and Per CP-conjugated anti-CD8 mAb; biotin-conjugated anti-rat IgG_2a_ and IgG_1_; streptavidin-phycoerythrin conjugate; appropriate isotype controls; and PE-conjugated annexin-V and 7-AAD (7-amino-actinomycin), were purchased from BD Phar Mingen (San Diego, CA, USA). For CD4+T cell stimulation functional grade anti-CD-3 and anti-CD28 Ab were purchased from eBioscience (ThermoFisher, Waltham, MA, USA). Silencer selected pre-designed GRAIL siRNA was purchased from Ambion (LifeTechnologies, Carlsbad, CA, USA), control siRNA from Santa Cruz Biotechnology and Lipofectamine and Lipofectamine RNAiMAX from Invitrogen (Carlsbad, CA, USA). Proteolipid protein PLP_139–151_ (HSLGKWLGHPDKF), and all peptides chosen for experiments were synthesized by the IBB PAN (Warsaw, Poland). PD10-columns, and ADP- and ATP- agarose were purchased from Sigma (St. Louis, MO, USA).

### 2.3. In Vitro Preparation of HINT1/Hsp70 Complexes

Pure unbound Hsp70 protein, was obtained from CNS tissues of healthy mice and purified according to previously published protocols [26]. Purified Hsp70 was complexed with synthetized HINT1 peptide (HINT1_38-57_ CLAFHDISPQAPHFLVIPK) as described previously [26]. The presence of Hsp70 and HINT1 in complex was confirmed by Western blot with anti-Hsp70 and anti-HINT1 antibody.

### 2.4. EAE Induction and Pretreatment with HINT1/Hsp70 Complex

6-week-old female SJL/J mice were pretreated by subcutaneous injection of HINT1/Hsp70 or control C3/Hsp70 complexes on day 14 and day 7 prior to sensitization for EAE with PLP_139–151_, as described previously [26]. EAE was induced with PLP_139–151_ peptide in CFA, containing 0.75 mg *M. tuberculosis*, one week after the last injection of HINT1/Hsp70 or C3/Hsp70 complex [26]. Mice were observed daily and scored as described before [26].

### 2.5. Magnetic Beads and FACS Cell Sorting

CD4+ T cells and NK cells were purified by magnetic beads sorting. CD4+ T lymphocytes were purified from spleen cells of EAE SJL/L or naïve C57BL6/J mice by negative magnetic beads sorting with CD4+ T Cell Isolation Kit (Miltenyi Biotec Inc., Auburn, CA, USA). Purity of CD4+ T cells was above 95%.

NK cells were negatively selected from spleen cells isolated from naïve mice or mice treated with HINT1/Hsp70 or C3/Hsp70 with magnetic beads by the depletion of non-NK cells using an NK Cell Isolation Kit (Miltenyi Biotec Inc., Auburn, CA, USA). Additionally, NK cells were purified by FACS sorting with anti-DX5 and anti-CD3ε antibody staining and the population positive for DX5 and negative for CD3ε was sorted out by a FACS Aria Cell Sorter (Becton Dickinson, Mountain View, CA, USA). Purity of NK cells was above 99%.

### 2.6. Flow Cytometry

NK cells and CD4+ T cells sorting efficiency was assessed by flow cytometry analysis. For assessment of NK-cell purity, NK cells were stained with FITC-conjugated rat anti-mouse CD49b/pan-NK mAb (DX5 Ab), PerCP-conjugated anti-CD3ε mAb. For assessment of CD4+T cell purity, CD4+ T cells were stained with FITC-conjugated anti CD4 mAb and PerCP-conjugated anti-CD3ε mAb.

For assessment of CD4+ T cell death, CD4+ cells isolated from EAE mice, stained with CFDA-SE, stimulated with PLP_139–151_ and cocultured with NK cells isolated from HINT1/Hsp70- treated mice for 3–5 days, after which cells were stained with annexin V conjugated to PE and 7-AAD and assessed by flow cytometry.

### 2.7. CD4+ T Cell Proliferation Assay

On day 14 after sensitization for EAE, CD4+ T cells were isolated from mice sensitized for EAE pretreated with HINT1/Hsp70 or from control non-pretreated mice. CD4+ T cells were stimulated with 50 µg/mL of PLP_139–151_, labeled with [^3^H] thymidine (Amersham, Biosciences, Buckinghamshire, UK) and harvested at 72 h. For assessment of proliferation of CD4+ T cells isolated from naïve C57BL6/J mice CD4+ T cells were stimulated with anti-CD3 and CD-28 antibody and labeled with [^3^H] thymidine (Amersham, Biosciences) and harvested at 72 h. Uptake was measured by liquid scintillation (Pharmacia, Uppsala, Sweden), and the stimulation index was calculated. For appropriate experiments CD4+ T cells after siRNA-GRAIL or control siRNA or pcDNA-GRAIL or control pcDNA3.1 transfections were used.

### 2.8. In Vitro Cell Cocultures

For coculture proliferation assays, NK cells were isolated from mice on day 14 after treatment with HINT1/Hsp70 or C3/Hsp70 as previously described [26] isolated NK cells were non-transfected or transfected with siRNA or pcDNA and then cocultured with PLP_139–151_-stimulated CD4+ T cells isolated from EAE SJL/J mice on day 14 post-PLP_139–151_ immunization, at a ratio of 1:4 as selected in previous experiments [26].

For NK cell transfection experiments, NK cells were isolated from the spleen cells of naïve mice (for pcDNA-GRAIL or control pcDNA3.1 transfection) or from the spleen cells of mice treated with HINT1/Hsp70 (for GRAIL siRNA transfection). For CD4+ T cell transfection experiments, CD4+ T cells were isolated from spleen cells of EAE mice or naïve C57BL6/J mice. All isolated cells for transfection experiments were divided into two fractions. The first was incubated with GRAIL siRNA or pcDNA-GRAIL, and the second with control siRNA or control pcDNA3.1. After transfection NK cells or CD4+ T cells were checked for transfection efficiency and used in coculture experiments for proliferation assessment.

### 2.9. Cell Transfection with siRNA or pcDNA Vector

NK cells from HINT1/Hsp70 mice were transfected with siRNA for GRAIL. Cells were incubated with GRAIL siRNA or control siRNA and Lipofectamine RNAiMAX for 24 and 48 h. Inhibition of expression of mRNA for GRAIL was checked by qRT-PCR. For further experiments, 48 h transfection was selected based on experiments assessing the inhibitory effect of siRNA. After siRNA transfection, NK cells were used in coculture experiments with PLP_139–151_ -stimulated CD4+ T lymphocytes isolated from spleen cells of EAE mice. Next, a proliferation assay was performed, as described above. Results were reported as the mean value of three independent experiments ±SD.

CD4+T cells isolated from spleen cells of EAE SJL/J mice pretreated with HINT1/Hsp70 were transfected with siRNA for GRAIL. Cells were incubated with GRAIL siRNA or control siRNA and Lipofectamine RNAiMAX for 24 and 48 h. Inhibition of expression of mRNA for GRAIL was checked by qRT-PCR. For further experiments, 48 h transfection was selected based on experiments assessing the inhibitory effect of siRNA. After siRNA transfection CD4+T cells were used in proliferation assay with PLP_139–151_ stimulation.

NK cells isolated from spleen cells of naïve mice were transfected with pcDNA-GRAIL or pcDNA3.1 control vector (Promega). Cells were incubated with 2 ug of pcDNA3.1 vectors and 1 ml lipofectamin, according to previous protocols [27]. Cells were grown for 48 h before usage in coculture experiments.

### 2.10. Cell Cytotoxicity

Cytotoxic activity of NK cells was measured by a Cytotoxicity Detection Kit (LDH, Roche Applied Science), according to the manufacturer’s protocol and previous experiments [26]. This assay allows for the quantification of cell lysis based on lactate dehydrogenase (LDH) release from damaged cells into the supernatant. For this purpose NK cells were cocultured for 4 h with CD4+ T cells stimulated with PLP_139–151_ at a ratio 1:4, and supernatants were collected. Additionally, target cells were lysed to assess total LDH release. Cytotoxicity was calculated as follows:

Cytotoxicity (%) = ((effector-target cell mix − effector cell control) − low control) × (high control − low control)^−1^ × 100

### 2.11. Gel Electrophoresis and Western Blot

For protein and peptide detection, samples were separated by SDS-PAGE and transferred to PVDF membrane for western blotting, as previously described [26].

### 2.12. qRT-PCR

Total RNA was isolated from FACS-sorted NK cells, obtained from naïve mice or mice pretreated with HINT1/Hsp70 or C3/Hsp70 complex, using RNeasy Mini Kit (Qiagen, Hilden, Germany) and from magnetic sorted CD4+ T cells isolated from EAE SJL/J or naïve C57BL6/J mice. Amount of RNA was measured by NanoDrop 2000 (ThermoScientific). Total RNA was transcribed into cDNA with High Capacity cDNA Reverse Transcription Kit (Applied Biosystems, Foster, CA, USA). cDNA was used for real-time PCR (qRT-PCR) assessment of GRAIL, DGKα, Egr2, Lag3 mRNA expression. For these, assessment qRT-PCR was performed with TaqMan^®^ probes stained with FAM (GRAIL) DGKα, Egr2, Lag3, beta-actin (mouse)) (Applied Biosystems) and TaqMan^®^ Gene Expression Master Mix (Applied Biosystems). All reactions were performed in triplicate using the 7500 RealTime PCR System (Applied Biosystems). Expression of mRNA for GRAIL, DGKα, Egr2, Lag3 mRNA expression were quantified by comparison with expression of the housekeeping gene.

### 2.13. GRAIL Vector Construction

To determine the function of GRAIL in CD4+ T cells and NK cells, a pcDNA3.1 (Promega, Madison, WI, USA) plasmid containing the GRAIL sequence in a sense orientation was constructed. The murine GRAIL sequence was taken from GenBank. The GRAIL sequences were copied from CD4+ T cells isolated from EAE mice preinjected with HINT1/Hsp70 using the primers 

mRnfREV1 5′ GCGCTCTAGATTAAGATTTAATCTCCCGAACAGCTGCCTCTTG3′ mRnfFOR1 5′ GAATGAATTCATGGGGCCGCCGCCCGGGAT3′ (IBB PAN, Warsaw, Poland) giving the PCR product a molecular weight of 1287 bp. PCR conditions were as follows: 95 °C, 5 min; 95 °C, 0.5 min; 68.5 °C, 1 min; 72 °C 2 min; 35 cycles, 72 °C, 10 min. The first PCR product was extracted from agarose gel and was cloned into a pcDNA3.1 vector in a sense orientation. The restriction sites/enzymes were EcoRI and XbaI. Vectors containing proper fragments were cloned using Escherichia coli DH5 strain of competent cells (Stratagene, San Diego, CA, USA). Plasmids were purified on Qiagen columns according to the manufacturer’s procedures (Qiagen). The transfection efficiency was assessed on RT-PCR by mRNA expression increase and on Western blot by increase of protein expression.

### 2.14. Statistical Methods

Data are expressed as means ± SD. For multiple comparison measures, Student’s *t*-test, ANOVA test were used.

## 3. Results

### 3.1. EAE Inhibition Induced by HINT1/Hsp70 Depends on NK Cells

**Figure 1:**
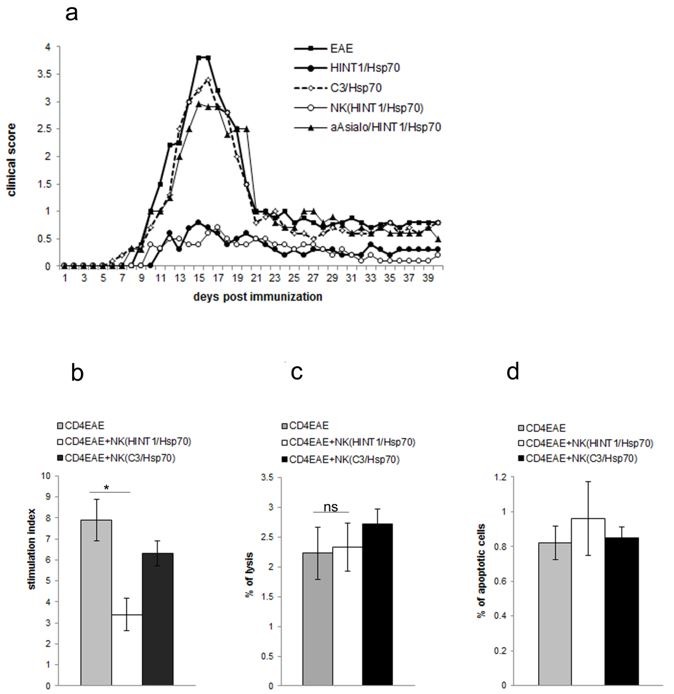
(**a**) The course of experimental autoimmune encephalomyelitis (EAE) was assessed in five groups of SJL/J mice: 1. non-pretreated (black squares); 2. pretreated with HINT1/Hsp70 complex (black circles); 3. pretreated with C3/Hsp70 complex (control group) (open diamond); 4. pretreated with HINT1/Hsp70 complex but also depleted of NK cells with anti-asialoGM1 Ab (black triangles); five with transfer of NK cells isolated from mice treated with HINT1/Hsp70 complex (but non-pretreated with HINT1/Hsp70 complex) (open circles). Mice were assessed by examining clinical score daily, using 0–5 scale. Each point represents the mean of the clinical course; Data are pooled from four independent experiments with six to eight mice per group (**b**) The PLP_139–151_-induced proliferation of CD4+ T cells isolated from EAE mice was assessed by [3H] thymidine uptake. CD4+ T cells isolated from EAE SJL/J mice were cocultured in a ratio of 4:1 with NK cells isolated from mice treated with HINT1/Hsp70 or C3/Hsp70 complex. Stimulation indices are shown as mean ± SD obtained from four independent experiments with three to five mice per experiment. * *p* < 0.001; (**c**) The cytotoxicity of NK cells to CD4+ T cells was measured by LDH release assay. NK cells isolated from mice treated with HINT1/Hsp70 or C3/Hsp70 were cocultured at a ratio of 1:4 with CD4+ T cells isolated from EAE mice and stimulated with PLP_139–151_. Data represent the mean percentage of lysis obtained from three independent experiments; ns - not significant; (**d**) The induction of apoptosis of CD4+ T cells isolated from EAE SJL/J mice induced by NK cells isolated from mice treated with HINT1/Hsp70 or C3/Hsp70. CD4+ T cells were labeled with CFDA-SE, stimulated with PLP_139–151_, and cocultured with NK cells for 120 h, then stained with annexin V–PE and 7-AAD, and assessed for presence of apoptotic cells by flow cytometry. Each bar represents the mean percentage of annexin V–PE and 7-AAD-positive PLP_139–151_ -responsive CD4+ T cells. Data were obtained from four independent experiments.

As we have shown previously [26], NK cells are indispensable in protection induced with HINT1/Hsp70 pretreatment. Pretreatment with HINT1/Hsp70 inhibited EAE development (Figure 1a) and this effect was abolished by NK cell depletion with anti-asialoGM1 antibody prior to HINT1/Hsp70 pretreatment and EAE induction. To further assess the role of NK cells stimulated with HINT1/Hsp70 we assessed CD4+ T cell proliferation in coculture experiments. The proliferation of CD4+ T cells isolated from EAE mice stimulated with PLP_139–151_ was inhibited by coculture with NK cells isolated from mice injected with HINT1/Hsp70 but not by NK cells isolated from C3/Hsp70 injected mice (control) (Figure 1b). This inhibitory effect of NK cells isolated from mice treated with HINT1/Hsp70 on PLP_139–151_ -induced proliferation of CD4+ T cells did not depend on cytotoxicity of NK cells as we have not seen necrosis or apoptosis of CD4+ T cells induced in co-culture system (Figure 1c,d).

### 3.2. GRAIL Expression in CD4+ T Cells Is Indispensable for Inhibition of CD4+ T Cells Proliferation

**Figure 2:**
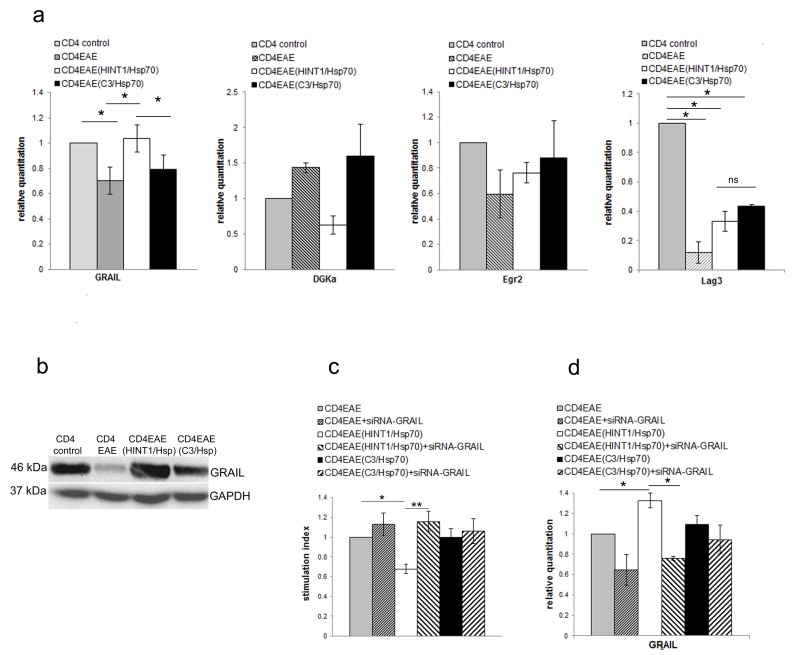
(**a**) Expression of mRNA for GRAIL, DGKα, Egr2 and Lag3 was measured with qRT-PCR in CD4+ T cells isolated from naïve SJL/J mice or EAE SJL/J mice non-pretreated or pretreated with HINT1/Hsp70 or C3/Hsp70. Ratio of mRNA was calculated versus the level of mRNA in CD4+ T cells isolated from naive mice. Data were pooled from three experiments with three to six mice per experiment. * *p* < 0.001; (**b**) GRAIL protein expression in CD4+ T cells isolated from EAE SJL/J mice non-pretreated or pretreated with HINT1/Hsp70 or C3/Hsp70 complex was analyzed by Western blot with usage of anti-GRAIL antibody. GAPDH was used as loading control. Blot is representative of three independent experiments; (**c**) The PLP_139–151_ peptide induced proliferation of CD4+ T cells isolated from EAE SJL/J mice non-pretreated or pretreated with HINT1/Hsp70 or C3/Hsp70 complex was assessed by [3H] thymidine uptake. Stimulation indices are shown as mean ± SD obtained from four independent experiments with five to six mice per experiment. * *p* < 0.001, ** *p* < 0.05; (**d**) Inhibition of GRAIL expression in CD4+ T cells isolated from EAE mice non-pretreated or pretreated with HINT1/Hsp70 or C3/Hsp70 by siRNA-GRAIL transfection. Efficacy of GRAIL mRNA inhibition with siRNA-GRAIL transfection was assessed by qRT-PCR. Ratio of mRNA was calculated versus the level of mRNA in CD4+ T cells isolated from non-pretreated EAE mice. Data are pooled from four independent experiments with five to six mice per experiment. * *p* < 0.001.

To further assess the inhibitory effect of NK cells on CD4+ T cells proliferation as it seemed to be not depended on cytotoxic effect the anergy related genes in CD4+ T cells were measured. The level of mRNA of GRAIL was significantly increased in CD4+ T cells but not DGKα, Egr2. (Figure 2a). In compare to CD4+ T cells isolated from naïve mice Lag3 was significantly downregulated in all other three groups of CD4+ T cells: 1. isolated from EAE mice, 2. isolated from EAE mice pretreated with HINT1/Hsp70, and 3. isolated from EAE mice pretreated with C3/Hsp70. These data might indicate that Lag3 downregulation is correlated with PLP immunization. There were no statistically significant differences in expression of Lag3 among CD4+ T cells isolated from EAE mice, EAE mice pretreated with HINT1/Hsp70 and EAE mice pretreated with C3/Hsp70. These data indicate that inhibition of EAE after HINT1/Hsp70 pretreatment cannot be related to Lag3 expression. Also GRAIL protein level was increased in CD4+ T cells isolated from EAE mice pretreated with HINT1/Hsp70 in comparison to CD4+ T cells isolated from not-pretreated EAE mice or EAE mice pretreated with C3/Hsp70 (Figure 2b). Additionally, transfection of CD4+ T cells isolated from EAE mice pretreated with HINT1/Hsp70 with siRNA-GRAIL reverse inhibitory effect of pretreatment with HINT1/Hsp70 on CD4+T cell proliferation induced with PLP_139–151_ (Figure 2c). The expression of GRAIL in CD4+ T cells was significantly reduced with siRNA-GRAIL transfection as assessed by RT-PCR (Figure 2d).

### 3.3. NK Cell Inhibitory Effect Depends on GRAIL Expression

**Figure 3:**
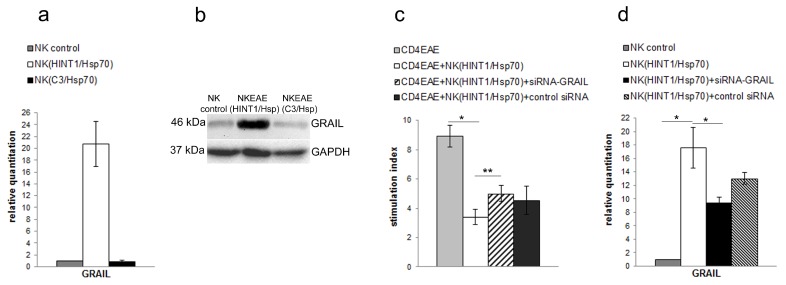
(**a**) Expression of mRNA for GRAIL was measured with qRT-PCR in NK cells isolated from naïve or HINT1/Hsp70 or C3/Hsp70 complex treated SJL/J mice. Ratio of mRNA was calculated versus the level of mRNA in NK cells isolated from naive mice. Data were pooled from four experiments with three to six mice per experiment; (**b**) GRAIL protein expression in NK cells isolated from naïve or HINT1/Hsp70 or C3/Hsp70 complex treated SJL/J mice was analyzed by Western blot with usage of anti-GRAIL antibody. GAPDH was used as loading control. Blot is representative of three independent experiments; (**c**) The PLP_139–151_ induced proliferation of CD4+ T cells isolated from EAE SJL/J mice were cocultured with three different types of NK cells isolated from SJL/J mice treated with HINT1/Hsp70 complex 1. non-transfected; 2 transfected with siRNA-GRAIL; and 3. transfected with control siRNA. After 72 h the proliferation was measured by [3H] thymidine uptake. Stimulation indices are shown as mean ± SD obtained from three independent experiments with three to six mice per experiment. * *p* < 0.001, ** *p*< 0.05; (**d**) Inhibition of mRNA GRAIL expression in NK cells isolated from SJL/J mice treated with HINT1/Hsp70 by siRNA-GRAIL transfection. Efficacy of GRAIL mRNA inhibition with siRNA-GRAIL transfection was assessed by qRT-PCR. The ratio of mRNA was calculated versus the level of mRNA in NK cells isolated from naïve SJL/J mice. Data are pooled from three independent experiments with five to six mice per experiment. * *p* < 0.001.

To further assess inhibitory effect of NK cells isolated from mice treated with HINT1/Hsp70 we assessed GRAIL expression in these cells. GRAIL was significantly increased in NK cells isolated from HINT1/Hsp70 treated mice assessed by qRT-PCR (Figure 3a) and Western blot (Figure 3b). To further assess GRAIL importance for inhibitory effect of NK cells on CD4+ T cells proliferation we decreased GRAIL expression by siRNA-GRAIL transfection and used them in coculture experiments with CD4+ T cells isolated from EAE mice. In these experiments, CD4+ T cells isolated from EAE mice were stimulated with PLP_139–151_ and cocultured with non-transfected or transfected with siRNA-GRAIL or control siRNA NK cells isolated from HINT1/Hsp70 treated mice. Reduction of GRAIL expression in NK cells isolated from mice treated with HINT1/Hsp70 significantly reduced inhibitory effect on CD4+ T cells proliferation (Figure 3c). However, the transfection of NK cells isolated from HINT1/Hsp70 mice with siRNA-GRAIL recover only partially the proliferation of CD4+ T cells. This only partial effect on CD4+ T cell proliferation might be explained by only partial reduction of GRAIL expression in NK cells transfected with siRNA-GRAIL what was confirmed by qRT-PCR (Figure 3d). The control siRNA transfection of NK cells isolated form HINT1/Hsp70 mice also affected GRAIL expression what might explain partial inhibitory effect of these cells on CD4+ T cell proliferation (Figure 3c,d).

**Figure 4:**
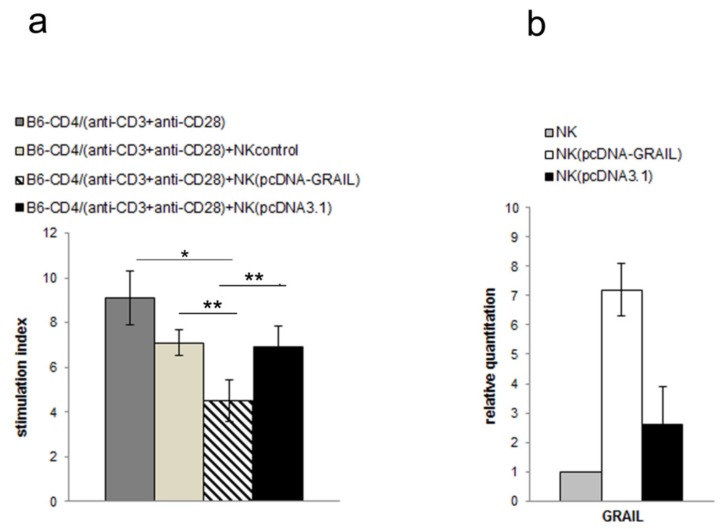
(**a**) The proliferation of anti-CD3 and anti-CD28 stimulated CD4+ T cells isolated from naïve C57BL6/J mice cocultured with NK cells isolated from naïve C57BL6/J mice non-transfected or transfected with pcDNA-GRAIL or pcDNA3.1 was assessed by [3H] thymidine uptake. Stimulation indices are shown as mean ± SD obtained from three independent experiments with three to six mice per experiment. * *p* < 0.001, ** *p* < 0.05; (**b**) Expression of mRNA GRAIL in NK cells isolated from C57BL6/J mice non-transfected or transfected with pcDNA-GRAIL or pcDNA3.1 was assessed by qRT-PCR. Ratio of mRNA was calculated versus the level of mRNA in NK cells isolated from naïve C57BL6/J mice. Data are pooled from three independent experiments with three to six mice per experiment.

To further confirm the importance of GRAIL for inhibitory effect on T cell proliferation we used NK cells isolated from C57BL6/J mice. Previously, we have shown that HINT1/Hsp70 induced protection depended on H60 expression [28]. The C57BL6/J mice did not express H60 and were resistant to protective effect of HINT1/Hsp70 pretreatment. Therefore, we have used NK cells isolated from naïve C57BL6/J mice and transfected them with GRAIL as we were not been able to induce GRAIL expression by HINT1/Hsp70 pretreatment to confirm GRAIL function in induction of tolerance dependent on NK cell. The NK cells isolated from naïve C57BL6/J mice transfected with GRAIL inhibited CD4+ T cell proliferation induced by anti-CD3 and anti-CD28 stimulation (Figure 4a) confirming GRAIL function in tolerance induction independent of manner of GRAIL overexpression. The increased GRAIL expression in NK cells isolated from C57BL6/J mice was confirmed by qRT-PCR (Figure 4b).

## 4. Discussion

Mechanisms underlying the regulation of immune responses still are not well known. In previous experiments, we showed that HINT1/Hsp70 complex induced tolerance to EAE [26,28,29]. The tolerogenic effect of this peptide complexed with Hsp70 was found to depend on activation of NK cells [29] and involved the NKG2D receptor and its ligand, H60 [29]. As we have shown previously and in present experiments, the regulatory role of NK cells did not depend on induction of necrosis or apoptosis of T cells [26]. In the present study, we have shown that HINT1/Hsp70 induced GRAIL expression in CD4+ T cells and this expression was essential for proliferation inhibition. The deletion of GRAIL in CD4+ T cells reversed inhibitory effect on T cell proliferation induced by PLP_139–151_ autoantigen. Therefore we proposed that HINT1/Hsp70 pretreatment induce CD4+ T cell anergy. The immunomodulatory treatments of MS affects T cell migration through blood-brain-barrier (natalizumab) or suppress T cell egress from lymph nodes (fingolimod) or delete cells in peripheral blood (alemtuzumab and ocrelizumab). However, cannabidiols used for treatment of spasticity in MS have been shown to induce T cell anergy depending on Egr2 expression [30]. This induction of anergy was accompanied by reduction in antigen presenting capacity by B cells [30].

Contradictory results regarding NK cell function in autoimmune demyelination have been obtained from studies conducted in EAE animal models. Mice deficient for fractalkine receptor expression, which is fundamental for the recruitment of NK cells in the CNS showed an increased EAE severity [24]. This evidence is supported by the investigation by Hao and colleagues [23] demonstrating inhibition of myelin-reactive Th17 by NK cells residing in the CNS. Shi and colleagues showed that NK cells augment the destructive process involving the CNS by the early release of IFN-ɣ [31]. Conversely, NK cell depletion at that early phase was able to consistently ameliorate EAE severity, support the pathogenic role played by NK cells [21].

As we have shown previously [26] and in present experiments, the inhibitory effect on EAE course exerted by HINT1/Hsp70 pretreatment depended on NK cells as depletion of NK cells with anti-asialoGM1 antibody resulted in augmentation of EAE. Similarly, NK cells isolated from HINT1/Hsp70 treated mice inhibited CD4+ T cell proliferation in vitro. Surprisingly this regulatory function of NK cells depended on GRAIL expression. Inhibition of GRAIL expression in NK cells isolated from HINT1/Hsp70 treated mice led to diminish inhibitory effect on T cell proliferation but not fully recover the proliferation of CD4+ T cells. This only partial effect on CD4+ T cell proliferation might be explained by only partial reduction of GRAIL expression in NK cells transfected with siRNA-GRAIL. The immunoregulatory function of HINT1/Hsp70 treatment depended on H60 expression [28]. This effect was not observed in C57BL6/J mice as H60 was not expressed in these mice. To confirm immunoregulatory function of GRAIL expressed in NK cells we transfected NK cells isolated from naïve C57BL6/J mice with GRAIL to overcome lack of possibility to induce this tolerogenic effect by HINT1/Hsp70 treatment. These NK cells transfected with GRAIL were able to inhibit CD4+T cell proliferation in vitro. The role of GRAIL in induction and maintenance of T cell anergy is well studied. However, the role of GRAIL in innate immune system is less well known. Lately, it was shown that GRAIL is also expressed in macrophages and is an essential positive regulator of antiviral immunity to RNA and DNA viruses. GRAIL was shown to be involved in regulation of IFNβ production. To our knowledge, there was no available reports on GRAIL expression and its function in NK cells. Most of data suggest that NK cells exert their immunoregulatory function by induction of cell death of activated T cells [19,20]. In our experiments, NK cells did not kill T cells but rather induce their anergy.

In summary, we have shown that HINT1/Hsp70 treatment generated regulatory NK cells expressing GRAIL indispensable for their inhibitory function. These NK cells induced anergy of autoreactive T cells. This novel immunoregulatory function of NK cells might have important implications in the designing treatment strategies for autoimmune diseases within the CNS including MS.
